# Effects of co-administered dexamethasone and diclofenac potassium on pain, swelling and trismus following third molar surgery

**DOI:** 10.1186/1746-160X-1-11

**Published:** 2005-11-07

**Authors:** Babatunde Olamide Bamgbose, Jelili Adisa Akinwande, Wasiu Lanre Adeyemo, Akinola Ladipo Ladeinde, Godwin Toyin Arotiba, Mobolanle Olugbemiga Ogunlewe

**Affiliations:** 1 Department of Oral and Maxillofacial Surgery, Lagos University Teaching Hospital, P.M.B 12003, Lagos, Nigeria; 2 Department of Oral and Maxillofacial Surgery, College of Medicine, University of Lagos, P.M.B 12003, Lagos, Nigeria

## Abstract

**Background:**

The apparent interactions between the mechanisms of action of non-steroidal anti-inflammatory drugs (NSAIDS) and steroids suggest that co-therapy may provide beneficial inflammatory and pain relief in the absence of side effects. The aim of the study was to compare the effect of co-administered dexamethasone and diclofenac potassium (diclofenac K) with diclofenac K alone on the postoperative pain, swelling and trismus after surgical removal of third molars.

**Patients and Methods:**

A prospective randomized double-blind study was conducted at the Department of Oral and Maxillofacial Surgery, Lagos University Teaching Hospital, Nigeria. A total of 100 patients were randomly allocated to two treatment groups of dexamethasone (prophylactic 8 mg and postoperative 4 mg IV) and diclofenac K (50 mg Oral before and after surgery), and diclofenac K alone (as with first group). The overall analgesic efficacy of the drug combinations was assessed postoperatively by determination of pain intensity using a category rating scale. Facial swelling was measured using a tape measure placed from tragus to gonion to tragus, while interincisal mouth-opening of patients was measured using a vernier calibrated caliper pre-operatively and post-operatively.

**Results:**

Co-administration of dexamethasone and diclofenac K was significantly superior to diclofenac alone for the relief of pain (P < 0.05), and facial swelling up to post-operative 48 hour (P < 0.05). However, there was no significant difference for trismus relief between the two medication protocols (P > 0.05).

**Conclusion:**

This study illustrates enhanced effects of co-administered dexamethasone and diclofenac K on short-term post-operative pain and swelling, compared to diclofenac potassium alone in third molar surgery.

## Introduction

Surgical removal of wisdom teeth under local anaesthesia is widely carried out in general dental practice and in many institutional surgery clinics, occupying an appreciable amount of clinical time [1,2]. This procedure is usually associated with postoperative pain, swelling, and trismus [1-4] as direct and immediate consequences of the surgical procedure [5,6]. The adverse effects of the wisdom tooth surgery on the quality of life has been reported to show a three-fold increase in patients who experience pain, swelling and trismus alone or in combinations; compared to those who were asymptomatic [5-7]. Many clinicians have, thus, emphasized the necessity for better pain, swelling and trismus control in patients who undergo third molar surgery [8,9].

The introduction of non-steroidal anti-inflammatory drugs (NSAIDs, e.g. diclofenac potassium and ibuprofen) has significantly altered the management of postoperative pain in dentistry and medicine. There are 2 possible mechanisms for the efficacy of NSAIDs when administered prior to surgical trauma. The first may simply be a pharmacokinetic advantage. By administering the NSAIDs prior to pain onset, drug absorption would have begun and therapeutic blood level will be present at the time of pain onset. Second, the presence of a cyclooxygenase inhibitor at the surgical site may limit the production of prostaglandins and prostacyclins associated with hyperalgesia and edema [10,11]. The use of corticosteroids (e.g. dexamethasone, betamethasone) is another preventive strategy for limiting postoperative edema and trismus following third molar extractions. Postoperative swelling and edema may be due in part to the conversion of phospholipids to arachidonic acid by phospholipase A_2, _and the resultant production of leukotrienes, prostacyclins, prostaglandins and thromboxane A_2, _acting as mediators of the inflammatory response. The use of steroids may inhibit the initial step in this process [12]. Clinical trials in oral surgery have also supported the hypothesis that preemptive NSAIDs and corticosteroids are effective in delaying and preventing many postoperative sequelae [10]. The apparent interactions between the mechanisms of action of non-steroidal anti-inflammatory drugs (NSAIDS) and steroids suggests that co-therapy may provide beneficial inflammatory and pain relief in the absence of side effects.

The aim of the study was, thus, to compare the effect of co-administered dexamethasone-diclofenac potassium with diclofenac potassium alone, on the postoperative management of pain, swelling and trismus following removal of impacted lower third molars.

## Patients and methods

Patients who attended the Oral and Maxillofacial surgery clinic of the Lagos University Teaching Hospital, requiring surgical removal of unilateral or bilateral (at least 15 days between the two surgical procedures), impacted mandibular third molar teeth under local anaesthesia were included. The study protocol and the informed consent forms were approved by the research and ethics committee of the hospital. The study protocol was explained to the patients in detail after which consent was obtained. Patients were randomly allocated into two groups in a double-blind fashion by using prepared randomizations in sealed envelopes. Criteria for exclusion of patients included: renal or hepatic disease, blood dyscrasia, previous or present gastric ulcers, heart disease, known hypersensitivities, allergies, or idiosyncratic reactions to any study medications, pregnancy and lactation. In addition, patients who had taken analgesics or anti-inflammatory drugs within 24 hours before surgery were excluded from the study. All selected candidates were free of pain and other inflammatory symptoms that included swelling, hyperemia and decreased mouth opening at the time of surgery.

In Group I, patients were given a combination dexamethasone and diclofenac potassium (diclofenac K). Group II comprised of patients who were given diclofenac K alone.

The degree of surgical difficulty was assessed using Winter's and Terence Ward lines and Pell-Gregory criteria [13]. Oral perioperative antibiotics (500 mg ampicillin-cloxacillin, SmithKline Beecham, England and 400 mg metronidazole, Aventis Pharm. Int., Switzerland) were administered to all patients 30 minutes before surgery.

### Operative procedure

Surgical extraction of the third molars was carried with buccal guttering technique after adequate elevation and reflection of buccal mucoperiosteal flap under local anaesthesia (2% lignocaine hydrochloride with 1:100,000 adrenaline). Tooth delivery was followed by meticulous irrigation of the surgical site with physiologic saline (0.9%). The three-sided mucoperiosteal flap was repositioned and sutured. A single operator performed all surgical procedures.

### Pain measurement

Preoperative pain was assessed using a four-point Category Rating Scale [14,15] Accordingly, pain was recorded as: "0-no pain" (patient experiences no discomfort), "1-mild pain" (almost unnoticeable pain), "2-moderate pain" (noticeable pain, but patient can still engage in routine daily activities), and "3-severe pain" (very noticeable pain which disturbs the patient's daily routine). For each patient, the appropriate score was recorded in the questionnaire by one operator at 48 h and by the patient on a daily basis for 5 days. Before leaving the clinic, the operator ensured that all patients were thoroughly instructed how to complete the pain self-assessment diary and when to take medications.

### Measurement of facial width

As no published method satisfies all criteria for assessing facial swelling, we decided to use a measuring tape to measure facial width and swelling in one-dimension only. Facial width (swelling) was measured using a measuring tape. The reference points used were the tip of tragus of left and right ears, with the gonium in between. A single operator, repeating the procedure three times on each patient, made the measurements. The average of measurements was then taken (in cm) and recorded. The measurements were carried out just before the surgery and at post-operative days 1, 2, and 7. Postoperative swelling was expressed as a percentage increase in facial width.

### Measurement of mouth-opening ability

A vernier-calibrated sliding caliper was used to measure maximum interincisal mouth-opening ability of the patient at the commencement of the procedure. The reference point used was incisal edge of the maxillary central incisor and incisal edge of mandibular central incisor at maximum opening available.

The measurements were made in triplicate and the average was recorded in millimetres (mm). The measurement was carried out just before the surgery and at post-operative days 1, 2, and 7. Postoperative trismus was measured as a percentage decrease in mouth opening.

### Medication

The operator supplied the medications to ensure compliance. Dexamethasone (Epil Pharmaceuticals, China) was given parenterally 8 mg 30 minutes preoperatively, and then 4 mg 6 hours postoperatively in two doses. Diclofenac K (Novartis, Switzerland) was given 50 mg, 30 minutes preoperatively; and thereafter 50 mg, 2 times daily for five days. All patients were placed on a five-day antibiotic regimen (500 mg Ampicillin-cloxacillin, SmithKline Beecham, England; 4 times daily and 400 mg Metronidazole, Aventis Switzerland, 3 times daily). All the medications were administered orally, except dexamethasone, which was administered parenterally.

### Statistical Analysis

Data was analyzed using SPSS for windows (v11.5, SPSS Inc, Chicago, IL) statistical software package. One-way analysis of variance, student's t-test and χ^2 ^were used for repeated measures for category rating scale, interincisal opening and facial swelling. The level of significance was set at *P *< 0.05.

## Results

A total of 100 patients (equally distributed into groups I and II) who completed the study were included in the analysis. The mean age of the participants was 27.9 ± 5.2 years (range, 19–45 years; group I: 29.8 ± 5.3 years and group II: 26.1 ± 4.5 years). The male-to-female ratio was 1:1.1. The radiographic analysis of the type of impactions showed that mesio-angular impaction constituted 51.0% of cases, followed by disto-angular impaction (21.0%, Table 1).

**Table 1:** Types of impactions

Types	Frequency (%)
Mesioangular	51 (51.0)
Distoangular	21 (21.0)
Horizontal	16(16.0)
Vertical	12(12.0)
Total	100 (100)

Table 2 presents postoperative pain intensity, facial swelling and maximal mouth opening on post-operative days 1 and 2. For group I, the mean pain score on days 1 and 2 was significantly lower than that for group II (p < .05, Table 2). Co-administration of Dexamethasone-diclofenac K led to a significant reduction in both postoperative pain and swelling on Days 1 and 2 when compared with diclofenac K alone (*P *< 0.05).

**Table 2:** Pain intensity, facial swelling and mouth opening at Days 1 and 2 (D1, D2) in group I (Dexamethasone-diclofecac K) and group II (Diclofenac only). Values are expressed as mean ± SD

Group	N	Pain intensity		Facial Swelling		Mouth Opening	
		D1	D2	D1	D2	D1	D2
Group I	50	0.62 ± 0.6*	0.5 ± 0.51*	30.9 ± 1.6*	31.0 ± 1.58*	36.0 ± 11.2	38.1 ± 10.05
Group II	50	1.64 ± 0.9*	1.3 ± 0.62*	31.7 ± 1.6*	32.04 ± 1.5*	39.2 ± 11.3	36.0 ± 10.02

*p < 0.05

Although there was no significant difference between the treatment groups with regard to reduction in mouth opening, the "trismus" scores of group I (0.31 mm) were lower than those of group II (3.19 mm) between 24 h and 48 h.

By the post-operative 7^th ^day, all symptoms had restored to the preoperative level in both groups. Neither groups demonstrated any adverse reaction, side effect or other complications (e.g., tendency for bleeding) during the follow-up period.

## Discussion

By pharmacologically controlling the extent of the inflammatory process, the intensity or severity of postoperative sequelae such as pain, swelling and trismus, may be reduced [16,17]. One technique that has been proposed for reduction of postoperative inflammation is the administration of corticosteroids [16]. Cortisol and the synthetic analogue of cortisol have the capacity to interfere with the physiologic processes of inflammation and, thus, suppress the development of local fever, redness, swelling and tenderness by which inflammation is recognized [16]. Another technique is to control the synthesis of prostaglandins. Prostaglandins play a major role in the induction of pain, inflammation, and fever [3,11]. The reduction of biosynthesis of prostaglandins by inhibition of the cyclo-oxygenase enzyme system is considered an important mechanism of action of NSAIDs. When administered preoperatively, NSAIDs have been shown to be particularly effective in combating postoperative pain [3,11].

Preventive strategies for postoperative management of pain and inflammation are based on the known ability of NSAIDs to block the arachidonic acid cascade. When NSAIDs are administered preoperatively, absorption and distribution of the medication may occur before the initiation of tissue trauma, the ensuing synthesis of prostaglandins and the subsequent inflammatory response. Prevention of the inflammatory response may decrease the sequelae of tissue trauma; especially the accompanying pain [11]. Diclofenac K has been shown to be useful in controlling postoperative pain after removal of third molars [18]. Diclofenac K is known to possess both analgesic and anti-inflammatory effect. Due to its anti-inflammatory effects [18], the administration of dexamethasone may synergize the anti-inflammatory effect of cataflam and contribute to the reduction of inflammatory exudates as well as edema and pain. Therefore the co-administration of diclofenac K and dexamethasone may be expected to reduce post-operative pain more than that achieved with diclofenac K alone [18].

The present study assessed the clinical effect dexamethasone-diclofenac K combination and diclofenac K alone on pain, facial swelling and trismus. Regardless of the drug combination used, the pattern of postoperative pain has been reported to increase between the post-operative days 1 and 3, after which the symptoms subside gradually within one week [19-22]. Our results confirm this observation. The comparison of pain intensity between the dexamethasone-diclofenac K group and diclofenac K group showed significant difference between the two groups (*P *< 0.05), indicating an enhanced analgesic effect of diclofenac K when administered in combination with dexamethasone. This finding corroborates with those of previous reports [3,23-26]. Schultze-Mosgau et al [25] investigated the combined use of ibuprofen and methylprednisolone for pain relief, concluding that this combination has good analgesic and anti-inflammatory action. It has also been reported that a single dose administration of a glucocorticoid reduces tissue levels of bradykinin and suppresses circulating levels of cortisol and beta-endorphin [25]. As known, bradykinin and kallidin are the two kinins that act independently as well as synergistically with products of the arachidonic acid cascade to produce both hyperalgesia as well as increased vascular permeability [26].

Post-surgical facial edema is difficult to quantify accurately, since it requires a three-dimensional measurement with an irregular, convex surface and can manifest itself internally as well as externally. Over the years, numerous researchers have tried various techniques in an effort to objectively measure edema [23,26], most of which are indirect assessments of the altered contours of skin surface. Measurement tools mentioned in the literature have included visual analog scales, trismus recordings, standardized stereo-radiographic or photographic measurements, computerized tomography, modified face bow devices, ultrasonography, facial plethysmographs, or various other means of taking direct facial measurements [23,26]. In the present study, a single measurement from the tip of tragus to gonion to the tip of contralateral tragus was taken. The recordings were made in triplicate and the average was recorded. It is noteworthy to mention herein that the cheek swelling following third molar surgery is diffuse in different planes and is very difficult to measure accurately. The co-administration of dexamethasone diclofenac K preoperatively and postoperatively, produced a clear reduction in postoperative pain and cheek swelling. The mean increase in facial swelling in days 1 and 2 in Group I (dexamethasone- diclofenac K combination) was significantly less than that of Group II (diclofenac K only). This result shows that co-administration of dexamethasone diclofenac K also enhances the control of postoperative facial swelling [24,27-29].

Independent T-test did not show any significant difference in reduction of mouth opening (trismus) between the study groups (*P *> 0.05). While this observation does not corroborate with those of previous reports [21,22,25], the enhanced effect of steroids on mouth-opening may be observed clinically. In the present study, the mean reduction in mouth-opening between the post-operative days 1 and 2 were 0.31 mm and 3.19 mm, for groups I (dexamethasone and diclofenac K) and II (diclofenac K only), respectively. This shows a "clinically significant" difference in the interincisal distance. These results indicate a positive clinical association between the adjunct use of dexamethasone and postoperative recovery of trismus in third molar surgery.

The time course for pain and facial swelling findings described in the present study are in agreement with those of a recent multicenter trial indicating similar symptoms that reached a maximum at Days 1 or 2 postoperatively and generally resolved at Day 7 [30,31].

The potency and dosage of dexamethasone within the first 24 h (total of 16 mg, including pre-operative dose) was adequate to enhance the efficacy of diclofenac K. It appears that steroids are preferably administered pre-operatively, extending the coverage up to 24–48 hours after surgery. Intravenous administration of dexamethasone, as done in the present study, enhances earlier bioavailability in comparison to oral administration [9]. Such treatment with high dosage does not impair adrenal function. Additionally, intravenous administration of dexamethasone prior to third molar surgery bears no detrimental impact on wound healing, even in patients predicted to be at high risk for delayed clinical recovery [9].

## Conclusion

This study illustrates enhanced effects of co-administered dexamethasone and diclofenac K on short-term post-operative pain and swelling, compared to diclofenac potassium alone in third molar surgery.

## Competing interests

The author(s) declare that they have no competing interests.

## Authors' contributions

BBO and JAA conceived the study. BBO and WLA coordinated the write-up, did literature search and submission of the article. ALL, GTA, and MOO participated in the writing of the manuscript. All the authors read and approved the final manuscript.

## References

[B1] Thomas D, Walker R, Smith A, Shepherd J (1994). The provision of oral surgery services in England and Wales 1984–1991. Br Dent J.

[B2] Antila H, Lehtinen R, Heinaro A, Lansineva A, Salonen M (1992). Successful Pain Management by Finnish Oral Surgeons. Oral Surg Oral Med Oral Pathol.

[B3] van der Westhuijzen AJ, Roelofse JA, Grotepass FW, Becker PJ (1994). Randomized double-blind comparison of tiaprofenic acid and diclophenac sodium after third molar surgery. Oral Surg Oral Med Oral Pathol.

[B4] Seymour RA, Kelly PJ, Hawkesford JE (1996). The efficacy of ketoprofen and paracetamol (acetaminophen) in post-operative pain after third molar surgery. Br J Clin Pharmacol.

[B5] Ruta DA, Bissias E, Ogston S, Ogden GR (2000). Assessing health outcomes after extraction of third molars: postopeartive symptom severity (PoSSe) scale. Br J Oral Maxillofac Surg.

[B6] McGrath C, Comfort MB, Lo ECM, Luo Y (2003). Changes in life quality following third molar surgery- the immediate postoperative period. Br Dent J.

[B7] Slade GD, Foy SP, Shugars DA, Phillips C, White RP (2004). The impact of third molar symptoms, pain and swelling on oral health-related quality of life. J Oral Maxillofac Surg.

[B8] Odgen GR (2003). Third molar surgery and postoperative pain relief. Br Dent J.

[B9] Tiwana PS, Foy SP, Shugars DA, Marciani RD, Conrad SM, Phillips C, White RP (2005). The impact of intravenous corticosteroid with third molar surgery in patients at high risk for delayed health-related quality of life and clinical recovery. J Oral Maxillofac Surg.

[B10] Moore PA, Brar P, Smiga ER, Costello BJ (2005). Preemptive rofecoxib and dexamethasone for prevention of pain and trismus following third molar surgery. Oral Surg Oral Med Oral Pathol Radiol Endod.

[B11] Jackson DL, Moore PA, Hargreaves KM (1989). Preoperative nonsteroidal anti-inflammatory medication for the prevention of postoperative dental pain. JADA.

[B12] Hirschman JV (1986). Some principles of systemic glucocorticoid therapy. Clin Exp Dermatol.

[B13] Akinwande JA (1991). Mandibular Third Molar Impaction- A comparison of two methods for predicting surgical difficulty. Niger Dent J.

[B14] Rodrigo MR, Rosenquist JB, Cheung LK (1987). Paracetamol and difflunisal for pain relief following third molar surgery in Hong Kong Chinese. Int J Oral Maxillofac Surg.

[B15] Ong KS, Seymour RA (2004). Pain Measurement in humans. Surg J R Coll Surg Edinb Irel.

[B16] Ito U, Reulen HJ, Tomita H, Ikeda J, Saito J, Maechara T (1998). Formation and propagation of brain edema fluid around human brain metastases. Acta Neurochirur (Wien).

[B17] Mense S (1981). Sensitization of group IV muscle receptor to bradykinin by 5-hydroxytryptamin and prostaglandin E2. Brain Res.

[B18] Matthews RW, Sully CM, Levers BGH (1984). The efficacy of diclofenac sodium with and without paracetamol in the control of postsurgical dental pain. Br Dent J.

[B19] Sisk AL, Bonnington GJ, Ga A (1985). Evaluation of methylprednisolone and flurbiprofen for inhibition of the postoperative inflammatory response. Oral Surg Oral Med Oral Pathol.

[B20] Seymour RA (1982). The use of pain scales in assessing the efficacy of analgesics in post-operative dental pain. Eur J Clin Pharmacol.

[B21] Hyrkas T, Ylipaavalniemi P, Oikarinen VJ, Paakkari I (1993). Pre-operative intravenous diclofenac for post-operative pain prevention in outpatients. Br J Oral and Maxillofac Surg.

[B22] Neupert EA, Lee JW, Philput CB, Gordon JR (1992). The evaluation of dexamethasone for reduction of postsurgical sequelae of third molar removal. J Oral Maxillofac Surg.

[B23] Roger EA, Roger RT (2000). A review of perioperative corticosteroid use in dentoalveolar surgery. Oral Surg Oral Med Oral Pathol.

[B24] Ross R, White CP (1958). Evaluation of hydrocortisone in prevention of postoperative complications after oral surgery: a preliminary report. Journal of Oral Surgery.

[B25] Schultze-Mosgau S, Schmelzeisen R, Frolich JC, Schmele H (1995). Use of ibuprofen and methylprednisolone for the prevention of pain and swelling after removal of impacted third molars. J Oral Maxillofac Surg.

[B26] Troullos ES, Hargreaves KM, Buttler DP, Dionne RA (1990). Comparison of nonsteroidal anti-inflammatory drugs, ibuprofen and flurbiprofen with methylprednisolone and placebo for acute pain, swelling and trismus. J Oral Maxillofac Surg.

[B27] Beirne OR, Hollander B (1986). The effect of methylprednisolone on pain, trismus, and swelling after removal of third molars. Oral Surg Oral Med Oral Pathol.

[B28] Beirne OR (1992). Evaluation of dexamethasone for reduction of postsurgical sequelae of third molar removal (Discussion). J Oral Maxillofac Surg.

[B29] Huffman G (1977). Use of methylprednisolone succinate to reduce postoperative edema after removal of impacted third molar. J Oral Surg.

[B30] White RP, Shugars DA, Shafer DM, Laskin DM, Buckley MJ, Philips C (2003). Recovery after third molar surgery: clinical and health-related quality of life outcomes. J Oral Maxillofac Surg.

[B31] Conrad SM, Blakey GH, Shugars DA, Marciani RD, Philips C, White RP (1999). Patients' perception of recovery after third molar surgery. J Oral Maxillofac Surg.

